# How stochastic adaptation of neurons shapes interspike interval statistics – theory and experiment

**DOI:** 10.1186/1471-2202-12-S1-P199

**Published:** 2011-07-18

**Authors:** Tilo Schwalger, Karin Fisch, Jan Benda, Benjamin Lindner

**Affiliations:** 1 Max Planck Institute for the Physics of Complex Systems, 01187 Dresden, Germany; 2 Department Biology II, Ludwig Maximilians University Munich, 82152 Planegg-Martinsried, Germany

## 

Trial-to-trial variability of neuronal responses is a prominent feature of sensory systems and has a significant impact on subsequent sensory signal processing. Fluctuations of the underlying ionic currents due to channel noise represent a major intrinsic source of noise that causes neuronal response variability. In many sensory systems it is, however, difficult to assess the type of channel noise by direct somatic recordings without severely damaging the sensory transduction machinery. Here we develop a novel indirect approach to distinguish different types of noise based on the interspike interval (ISI) statistics. The method is then used to determine the dominating source of noise of a sensory neuron. In particular, we seek to distinguish noise originating from fast spike-generating channels from channel noise associated to slow adaptation currents. Adaptation currents are found in many neurons and profoundly shape the signal transmission properties through the phenomenon of spike-frequency adaptation.

**Figure 1:**
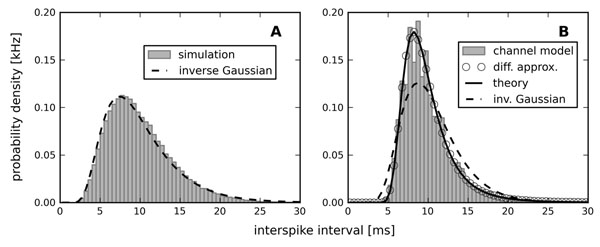
Interspike interval densities of adaptive integrate-and-fire model. Inverse Gaussian distribution fits for a deterministic adaptation current (A), but not for a stochastic adaptation current (B).

The effect of fast channel noise and slow adaptation channel noise is studied separately in an integrate-and-fire model augmented by an adaptation current. We show by means of analytical techniques that the shape of the ISI histograms and the ISI serial correlations are markedly different in both cases: for a deterministic adaptation current and fast noise, ISIs are distributed according to an inverse Gaussian density and the serial correlations are negative. In contrast, for stochastic adaptation currents, the ISI density is strongly peaked compared to an inverse Gaussian density (Figure 1) and the serial correlations are positive. In general, the calculation of serial correlation coefficients and higher-order ISI cumulants in the presence of adaptation and noise is a hard theoretical problem, because the model is non-renewal. Here, we put forward several novel analytical results for these measures [1].

We applied these measures to intracellular recordings of auditory nerve fibers of Locusta migratoria during simultaneous acoustic stimulation with pure tones of various intensities. The auditory receptors exhibit spike-frequency adaptation and the steady-state ISI statistics show a high variability with CVs up to 0.9 depending on sound intensity. With increasing spike frequency the shape of the ISI histograms changes from an inverse Gaussian to a peaked probability density. Additionally, the ISI correlations exhibit a shift from slightly negative values to positive coefficients with increasing spike rate. These observations can be indeed expected from and explained by our theoretical model. This indicates that stochasticity of slow adaptation currents may contribute to neural variability in sensory neurons.
